# Fosfomycin Disk Diffusion Testing among Klebsiella pneumoniae Results in Frequent Inner Colonies and Categorical Disagreement Based on Conflicting Breakpoint Organization Recommendations

**DOI:** 10.1128/spectrum.03363-22

**Published:** 2023-03-06

**Authors:** Morgan L. Bixby, Jenna M. Salay, Amanda R. Krueger, Amy J. Mathers, Elizabeth B. Hirsch

**Affiliations:** a University of Minnesota College of Pharmacy, Minneapolis, Minnesota, USA; b Department of Medicine and Pathology, University of Virginia, Charlottesville, Virginia, USA; Hartford Hospital

**Keywords:** fosfomycin, disk diffusion, inner colonies, agreement, interpretation, *Klebsiella pneumoniae*

## Abstract

Recent studies indicate that discrete inner colonies (ICs) arise during fosfomycin disk diffusion (DD) testing. CLSI and EUCAST have contradicting recommendations on the interpretation of ICs; CLSI recommends considering them while EUCAST recommends ignoring them when interpreting DD results. We sought to compare the categorical agreement of DD and agar dilution (AD) MIC and to assess the implications of ICs interpretation on zone diameter readings. A convenience sample of 80 Klebsiella pneumoniae clinical isolates with varied phenotypic profiles collected from 3 United States locations was included. Susceptibility was determined in duplicate, using both organization recommendations and interpretations for *Enterobacterales*. Correlations between methods were calculated using EUCAST_IV_ AD as the reference method. MIC values ranged from 1 to >256 μg/mL with an MIC_50/90_ of 32/256 μg/mL. Extrapolating EUCAST_oral_ and CLSI AD Escherichia coli breakpoints, 12.5% and 83.8% of isolates were susceptible, respectively, whereas 66.3% were susceptible by EUCAST_IV_ AD—which applies to K. pneumoniae. CLSI DD measurements were 2 to 13 mm smaller than EUCAST measurements due to 66 (82.5%) isolates producing discrete ICs. Categorical agreement with EUCAST_IV_ AD was greatest for CLSI AD (65.0%) and poorest for EUCAST_oral_ DD (6.3%). Isolates among this collection were frequently classified into different interpretive categories based on varying breakpoint organization recommendations. The more conservative oral breakpoints of EUCAST resulted in more isolates categorized as resistant despite frequent ICs. Differing zone diameter distributions and poor categorical agreement highlight issues of extrapolating E. coli breakpoints and methods to other *Enterobacterales*, and the clinical relevance of this issue warrants further investigation.

**IMPORTANCE** Fosfomycin susceptibility testing recommendations are complex. Both the Clinical and Laboratory Standards Institute and the European Committee on Antimicrobial Susceptibility Testing (EUCAST) recognize agar dilution as the reference method, but they also support disk diffusion as an approved method for Escherichia coli. However, these two organizations have conflicting recommendations for the interpretation of inner colonies that arise during disk diffusion testing which can lead to varying zone diameters and interpretations despite isolates having identical MIC values. Using a collection of 80 Klebsiella pneumoniae isolates, we found that a large (82.5%) portion produced discrete inner colonies during disk diffusion and isolates were frequently classified into different interpretive categories. The more conservative breakpoints of EUCAST resulted in more isolates categorized as resistant despite frequent inner colonies. Differing zone diameter distributions and poor categorical agreement highlight issues of extrapolating E. coli breakpoints and methods to other *Enterobacterales*, and the clinical relevance of this issue warrants further investigation.

## INTRODUCTION

Fosfomycin is an old antibiotic that was developed in the 1960s and approved by the United States Food and Drug Administration (FDA) in 1996 (1, 2). It has a broad spectrum of activity which includes activity against multidrug-resistant (MDR) Gram-negative pathogens, such as extended-spectrum β-lactamase (ESBL)-producing organisms and carbapenem-resistant *Enterobacterales* (CRE) (3, 4). Oral fosfomycin is a first-line antibiotic recommended for the treatment of uncomplicated urinary tract infections (UTI) and is approved only for the treatment of UTI caused by Escherichia coli or Enterococcus faecalis; however, it is often used to treat UTI caused by non-E. coli organisms as there are limited oral antibiotics available to treat UTI caused by increasingly resistant urinary pathogens (5–9). Clinical outcome data have demonstrated mixed results for the treatment of MDR infections, and most retrospective analyses for the treatment of UTI with oral fosfomycin are difficult to interpret since follow-up is not routine in these studies (5, 6, 8).

Fosfomycin susceptibility testing recommendations are complex. Both the Clinical and Laboratory Standards Institute (CLSI) and the European Committee on Antimicrobial Susceptibility Testing (EUCAST) recognize agar dilution (AD) as the reference method for fosfomycin susceptibility testing, but they also support disk diffusion (DD) as an approved testing method for E. coli isolates (10, 11). AD is both a labor- and time-intensive method and is not a feasible option for many clinical microbiology laboratories, so DD is often utilized in clinical settings. Since the *Enterobacterales* utilize two main nutrient transport systems responsible for fosfomycin uptake, one of whose expression, UhpT, is induced by glucose-6-phosphate (G6P), supplementation with G6P is also recommended for all fosfomycin testing methods (12). Finally, oral fosfomycin breakpoints for Gram-negative organisms from both CLSI and EUCAST are currently established only for E. coli and thus breakpoints for any non-E. coli *Enterobacterales* must be extrapolated, which is currently unsupported (10, 11, 13, 14). EUCAST has additional AD breakpoints for the intravenous (i.v.) formulation of fosfomycin that apply to all *Enterobacterales*, including K. pneumoniae; however, there are no equivalent CLSI breakpoints for i.v. fosfomycin (10, 11).

CLSI and EUCAST have conflicting recommendations for the interpretation of inner colonies that arise during fosfomycin DD testing despite the use of the same disk mass (200 μg with 50 μg G6P). CLSI states that the zone of inhibition shall be measured from the innermost colonies, whereas EUCAST states that the inner colonies shall be ignored (10, 15). Several recent fosfomycin studies have highlighted the observation of inner colonies within the zone of inhibition during fosfomycin disk diffusion testing (14, 16–18). The presence of these inner colonies present during within Etest inner inhibition zones has been linked to (nonfosfomycin) antibiotic heteroresistant phenotypes in other organisms (19). The difference in recommendations for interpreting DD results between organizations can lead to isolates with the same MIC having varying zone diameters, ultimately resulting in different susceptibility interpretations. In 2018, a CLSI *ad hoc* working group reviewed data surrounding these DD inner colonies. It was determined that the CLSI would not amend their methods because colonies within the zone were determined to be rare for E. coli, there was a lack of clinical outcome data, and there were concerns that amending this method might imply that colonies could be ignored for other non-E. coli organisms where additional resistance genes (e.g., *fosA*) could be attributed to their occurrence (https://www.idsociety.org/idsa-newsletter/september-26-2018/clsi-updates-from-the-clsi-subcommittee-on-susceptibility-testing).

Finally, the two organizations have considerably different breakpoints, with EUCAST having more conservative zone diameter measurements and lower MIC breakpoints, especially for the oral formulation of fosfomycin (10, 11). In 2020, EUCAST revised their oral fosfomycin breakpoints to reduce the MIC resistance threshold from >32 μg/mL down to >8 μg/mL, and these breakpoints were restricted for use only in E. coli from their previous approval for all *Enterobacterales* (20). However, fosfomycin breakpoints have not been reviewed by CLSI again since the EUCAST change.

In light of these differing breakpoints and contradictory inner colony interpretive criteria published by CLSI and EUCAST that are being extrapolated for use in non-E. coli *Enterobacterales*, we sought to compare categorical agreement of DD and AD between the extrapolated breakpoints from CLSI and EUCAST oral breakpoints to the approved EUCAST i.v. breakpoints. Additionally, we sought to assess the implications of the inner colony interpretation on zone diameter readings among a convenience collection of 80 clinical K. pneumoniae isolates.

## RESULTS

### Susceptibility testing.

Using AD, the MIC range and MIC_50/90_ remained the same between both organizations, with a range of 1 to >256 μg/mL and MIC_50/90_ of 32/256 μg/mL. The resulting interpretive categories of the isolates varied based on the testing procedures and which organizations’ breakpoints were applied or extrapolated for use (Table 1). When using AD, 66.3% (*n* = 53) isolates were considered susceptible when applying EUCAST i.v. breakpoints, compared with 83.8% (*n* = 67) when extrapolating CLSI breakpoints, and 12.5% (*n* = 10) when extrapolating EUCAST_oral_ breakpoints (Table 2).

**TABLE 1:** CLSI and EUCAST breakpoints for oral and intravenous fosfomycin

Parameter	MIC (μg/mL) by category			Zone diam (mm) by category		
	Susceptible	Intermediate	Resistant	Susceptible	Intermediate	Resistant
EUCAST_oral_^*a*^	≤8	NA^*b*^	>8	≥24	NA	<24
EUCAST_IV_	≤32	NA	>32	≥21^*c*^	NA	<21^*c*^
CLSI^*a*^	≤64	128	≥256	≥16	13–15	≤12

a All breakpoints apply only to E. coli.

b NA, not available.

c Zone diameter breakpoints apply only to E. coli.

**TABLE 2:** Comparison of susceptibility for agar dilution and disk diffusion using both CLSI and EUCAST breakpoints

Parameter	Values (*n* [%]) by category			MIC/zone range^*a*^	MIC_50/90_^*a*^
	Susceptible (S)	Intermediate (I)	Resistant (R)		
EUCAST oral breakpoints^*b*^ (*n* = 80)					
Agar dilution	10 (12.5)	NA	70 (87.5)	1 to >256	32/256
Disk diffusion	5 (6.3)	NA	75 (93.8)	6 to 30	20/23
EUCAST intravenous breakpoints (*n* = 80)					
Agar dilution^*c*^	53 (66.3)	NA	27 (33.7)	1 to >256	32/256
Disk diffusion^*b*^	28 (35.0)	NA	52 (65.0)	6 to 30	20/23
CLSI breakpoints^*b*^					
Agar dilution	67 (83.8)	3 (3.7)	10 (12.5)	1 to >256	32/256
Disk diffusion	34 (42.5)	20 (25.0)	26 (32.5)	6 to 2	15/20

a Units for values for agar dilution are μg/mL and those for disk diffusion are mm.

b Breakpoints apply only to E. coli.

c Approved breakpoint for all *Enterobacterales*, including K. pneumoniae.

**FIG 1:**
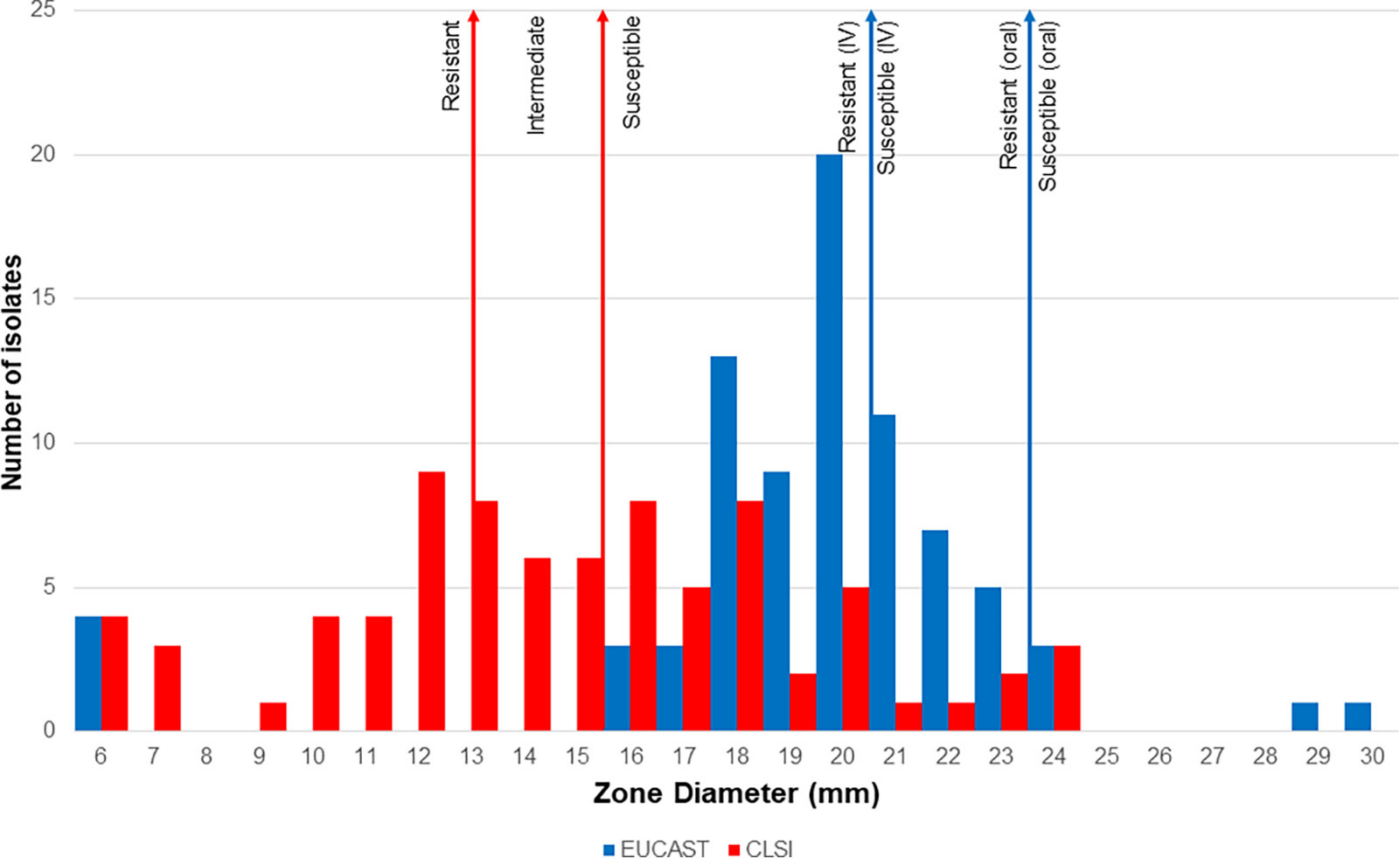
Zone diameters measured using both CLSI and EUCAST disk diffusion protocols for 80 K. pneumoniae isolates.

When using DD per each organization’s standard operating procedures (SOPs) for interpreting the results of DD (per E. coli breakpoints), 42.5% (*n* = 34) were susceptible per CLSI and 35.0% per EUCAST_IV_, while only 6.3% (*n* = 5) were susceptible per EUCAST_oral_. A total of 66 (82.5%) isolates displayed discrete inner colonies during at least one replicate of DD testing, accounting for differences in zone diameter measurements based on SOP recommendations. A majority (*n* = 42) of isolates had at least five discrete ICs. The zones of inhibition for this isolate set ranged from 6 to 24 mm using CLSI and 6 to 30 mm using EUCAST SOP (Fig. 1). When utilizing the CLSI DD SOP, the median zone of inhibition was 15 mm (considered intermediate [I], 13 to 15 mm). When utilizing the EUCAST DD SOP, the collection had a median zone of inhibition of 20 mm (considered resistant [R_oral_], <24 mm; R_IV_, <21 mm]).

**FIG 2:**
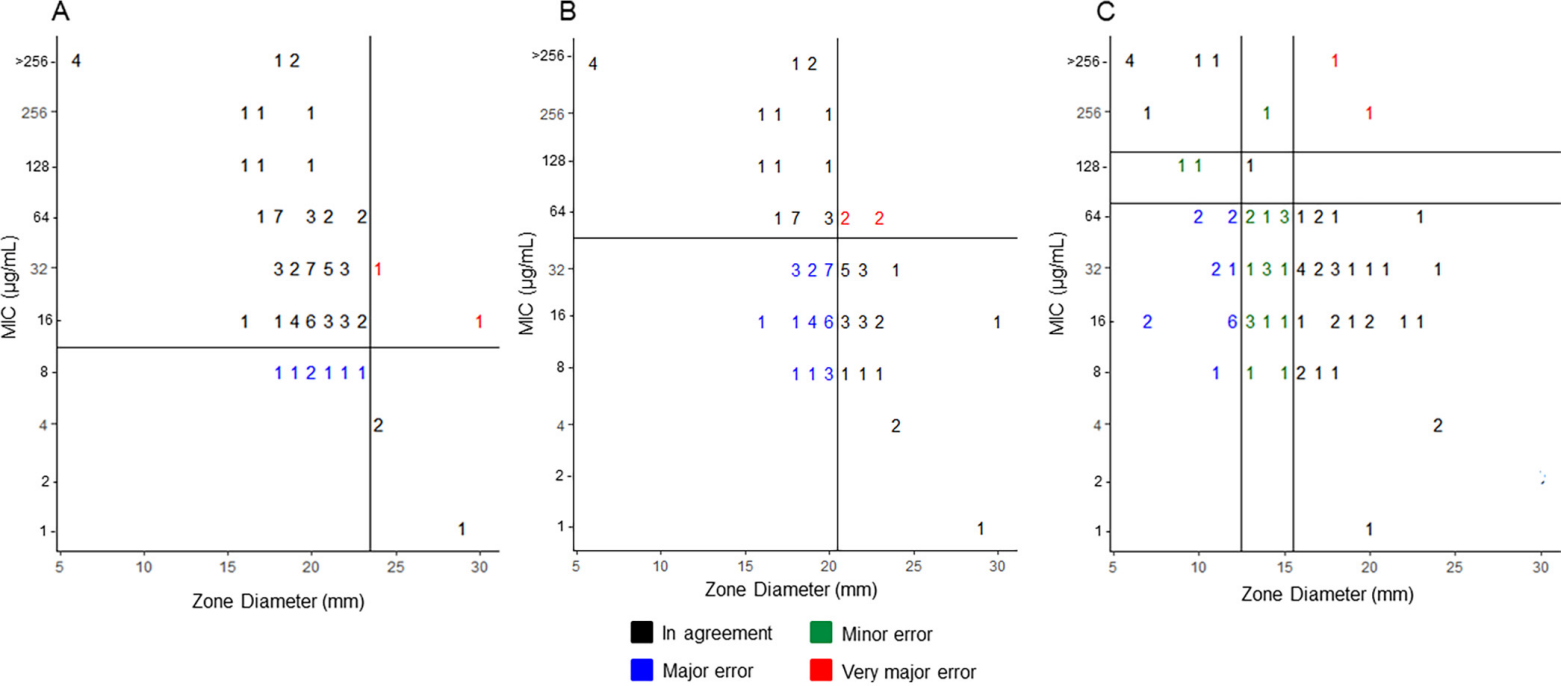
Scattergram comparisons of K. pneumoniae isolates according to fosfomycin zone diameter measurements and MIC values using agar dilution and read according to EUCAST and CLSI recommendations. (A) EUCAST_oral_. EUCAST zone diameter SOP was used for measurements, and horizontal and vertical lines are extrapolated EUCAST_oral_ E. coli breakpoints. (B) EUCAST_IV_. EUCAST zone diameter SOP was used for measurements, the horizontal line is the applied *Enterobacterales* MIC breakpoint, and the vertical line is the extrapolated EUCAST_IV_ E. coli breakpoint. (C) CLSI. CLSI zone diameter SOP was used for measurements, and horizontal and vertical lines are extrapolated CLSI E. coli breakpoints.

Using the EUCAST procedure for DD, when inner colonies were present, the zone of inhibition was 2 to 13 mm larger than CLSI measurements on the same plate, with a median difference of 4 mm. When inner colonies were present, zone diameter distributions followed visually distinct patterns when comparing measurements (Fig. 1). EUCAST data clustered near the median zone measurement (20 mm) with the majority of the isolates in this collection having a zone of inhibition around that peak (16 to 24 mm), with few isolates having a zone of inhibition outside that range. However, the CLSI data had a more even distribution across zone measurements. Additionally, a comparison of the zone diameters per each organization’s SOP for individual MIC values shows a greater number of zone diameters measured using the CLSI SOP than that using EUCAST (Fig. 2). EUCAST SOP (Fig. 2A and B) resulted in more isolates with the same MIC sharing a zone diameter measurement than CLSI (Fig. 2C) where there were a greater amount of isolates that did not share a zone diameter measurement with another isolate at that MIC.

### Correlation of susceptibility testing methods.

Categorical agreements calculated showed that the greatest agreement was with CLSI AD (65.0%, 52/80 isolates) where 3.8% (n/N, 3/80) of the isolates had minor errors and 57.7% (n/N, 15/26) of the resistant isolates had very major errors due to the one-dilution difference in susceptible breakpoints (Table 3). The poorest categorical agreement was with EUCAST_oral_ DD (6.3%, 5/80 isolates), where 90.4% (n/N, 47/52) of susceptible isolates had major errors and no resistant isolates had very major errors.

**TABLE 3:** Agreement between CLSI and EUCAST susceptibility testing results compared with EUCAST AD i.v. as the reference method

Method	Categorical agreement results (*n* [%])	No. (%) of isolates with:		
		Minor error	Major error	Very major error
EUCAST_IV_ DD	48/80 (60.0)	NA	29/52 (55.8)	4/26 (15.4)
EUCAST_oral_ AD	10/80 (12.5)	NA	42/52 (80.8)	0/26 (0.0)
EUCAST_oral_ DD	5/80 (6.3)	NA	47/52 (90.4)	0/26 (0.0)
CLSI AD	52/80 (65.0)	3/80 (3.8)	0/52 (0.0)	15/26 (57.7)
CLSI DD	35/80 (43.8)	20/80 (25.0)	12/52 (22.2)	7/26 (26.9)

## DISCUSSION

Several recent fosfomycin studies have highlighted the observation of inner colonies within the zone of inhibition during fosfomycin disk diffusion testing (14, 16–18). However, conflicting recommendations for the interpretation of these colonies from CLSI and EUCAST can lead to various zone diameters and susceptibility interpretations despite isolates having identical MIC values.

This study sought to compare the susceptibility of a collection of K. pneumoniae isolates (*n* = 80) between CLSI and EUCAST procedures and breakpoints for both AD and DD testing. The results were distributed widely where 6.3 to 83.8% of the isolates were considered susceptible per the currently established CLSI and EUCAST_oral_ breakpoints for E. coli and the EUCAST_IV_ breakpoints for all *Enterobacterales*. The CLSI AD breakpoints resulted in the most isolates being categorized as susceptible, and EUCAST_oral_ DD resulted in the most isolates being categorized as resistant. This finding is a result of EUCAST having a lower oral susceptibility breakpoint MIC (≤8 μg/mL) than their i.v. (≤32 μg/mL) and CLSI (≤64 μg/mL) breakpoints and the difference in zone diameter breakpoints. Additionally, a large proportion of isolates displayed inner colonies during DD testing (82.5%) resulting in remarkably different zone diameter measurements between CLSI and EUCAST before extrapolating their different interpretive categories. The practice of taking inner colonies into account when measuring the zone of inhibition resulted in greater inconsistencies in DD measurements for isolates with identical MIC values. Thus, the zone diameter measurements followed different distributions, indicating that the differing interpretation of ICs, when present, affects the susceptibility categories.

When comparing CLSI AD and EUCAST_oral_ AD to EUCAST_IV_ AD, there was greater agreement with CLSI (65.0%) than EUCAST_oral_ (12.5%). This result is strictly a reflection of the single 2-fold dilution difference between the reference EUCAST_IV_ susceptible breakpoint and CLSI compared with the two 2-fold dilution difference in the MIC breakpoints between the two EUCAST breakpoints. Additionally, the greatest agreement between any DD methods and breakpoints compared with the reference of EUCAST_IV_ AD was EUCAST_IV_ DD (60.0%), followed by CLSI (42.8%), and the worst was EUCAST_oral_ DD (6.3%), albeit none of these agreements are strong and have large amounts of major (22.2 to 90.4%) and very major (15.4 to 26.29%) errors. The error rates exceeded the CLSI published acceptable error rates of <10% minor errors, 3% major errors, and 1.5% very major errors (21, 22). This finding can be attributed to both the difference in breakpoints and the difference in zone measurements from the two organizations for the 82.5% of isolates where ICs arose during DD testing.

Mojica et al. (18) recently conducted a similar analysis of K. pneumoniae isolates (*n* = 68) collected in Colombia; however, all isolates were resistant to third-generation cephalosporins or carbapenems. They reported 90% of isolates to be susceptible per CLSI DD, 99% per CLSI AD, and 62% per EUCAST DD. This analysis was completed prior to EUCAST’s AD breakpoint change (from 32 μg/mL to 8 μg/mL); therefore, a smaller amount of the 96% of isolates considered susceptible per EUCAST AD with the current breakpoint applied. Interestingly, the authors reported a much lower incidence (19%) of inner colonies whereas we saw them arise in 82.5% of our isolate collection (18). Similar to our results, Elliott et al. (16) recently noted that inner colonies were “frequently present” for non-E. coli species during fosfomycin DD testing among a collection of Klebsiella pneumoniae carbapenemase (KPC)-producing isolates. All KPC-producing K. pneumoniae isolates (*n* = 24) carried *fosA* per whole-genome sequencing. It is likely that most clinical K. pneumoniae isolates carry fosA and play a role in the formation of inner colonies. Even though our collection included isolates with susceptible phenotypes (*n* = 36), the MIC_50/90_ values of 32/256 μg/mL were slightly higher than those reported (8/32 μg/mL) by Mojica et al. (18).

Furthermore, Abbott et al. (23) reported a presence of *fosA* in all their K. pneumoniae isolates (*n* = 20) with 60% (*n* = 12) of those screening positive for heteroresistance using a proposed disk elution method. Using an *in vitro* bladder infection model, the same authors found that baseline high-level heteroresistance in K. pneumoniae was associated with regrowth or treatment failure even when isolates had MICs that were lower than or equivalent to nonheteroresistant E. coli (24, 25). An evaluation of the correlation between heteroresistance and ICs presence among our collection is currently being conducted.

Lucas et al. (17) have also noted the presence of inner colonies in E. coli isolates during disk diffusion but at a much lower incidence (8%) than the K. pneumoniae studies. This study was able to attribute some amount of the IC production to the presence of mutations in *uhpT*, which encodes the nutrient transport pump UhpT. Such mutations cause increased resistance to fosfomycin through reduced function in or loss of a functional UhpT pump which also reduced fosfomycin uptake. However, this increased resistance is believed to come at a fitness cost to the bacterial cells because they are less able or unable to utilize G6P as a nutrient with an altered UhpT pump; it is unknown how reliably these results can be applied to other non-E. coli *Enterobacterales* (17, 26).

One of the strengths of this study is the inclusion of the measurement of zone diameter SOPs of both organizations, as they result in different measurements when inner colonies are present. The inclusion of an isolate set with diverse phenotypes collected from multiple geographic areas is also a strength of our work. However, this study is limited by its sample size as it is too small to perform an accurate epidemiological cutoff (ECOFF) value analysis or suggest appropriate breakpoints for K. pneumoniae. However, the EUCAST MIC Distributions for Fosfomycin database (retrieved 19 August 2022) displays the MIC distribution for 1,396 K. pneumoniae isolates which follow a similar MIC distribution (ECOFF, 128 μg/mL) to the isolate collection in the current study (https://mic.eucast.org/search/). An analysis of genomic resistance mechanisms for the isolates producing inner colonies is under way.

In conclusion, our data suggest that CLSI and EUCAST would frequently classify K. pneumoniae isolates with the same fosfomycin MIC into different interpretive categories based on the use of AD and DD, as well as based on the SOPs of the organizations. Our data also show that the interpretation of ICs greatly affects the resulting zone diameter and the interpretive category of the isolate, which supports both EUCAST and CLSI current recommendations that DD is an unsupported method for non-E. coli organisms at the current breakpoints. The inconsistent categorization of isolates with identical MIC based on various organizations’ breakpoints and methods necessitates further studies to determine the clinical importance, most appropriate interpretation of inner colonies present during fosfomycin DD testing, and hazards of extrapolating to non-E. coli *Enterobacterales*.

## MATERIALS AND METHODS

### Bacterial isolates.

This study included a convenience sample of 80 K. pneumoniae clinical isolates collected from 3 U.S. locations from 2013 to 2016 (27–29). The anatomical culture sites of the isolates included urine (*n* = 63), blood (*n* = 9), sputum (*n* = 5), and wounds (*n* = 3). Phenotypic susceptibility profiles varied and included 36 isolates with susceptible phenotypes, 21 confirmed extended-spectrum beta-lactamase (ESBL)-producing isolates, 11 Klebsiella pneumoniae carbapenemase (KPC)-2-producing isolates, 8 isolates resistant to third/fourth-generation cephalosporins (unconfirmed ESBL status), and 4 KPC-3-producing isolates (27–29).

### Susceptibility testing and disk diffusion interpretation.

Fosfomycin susceptibility was determined in duplicate, on separate days, via AD and DD using both CLSI and EUCAST procedures (10, 11, 15). When disk diffusion was performed, for which both organizations state that the methods and breakpoints apply only to E. coli, two measurements were collected (i) following the CLSI procedures to measure the innermost zone of inhibition when a minimum of one single discrete inner colony was present and (ii) following EUCAST procedures to measure the outermost zone of inhibition, ignoring the presence of any discrete inner colonies when present.

Test isolates were inoculated onto blood agar plates for overnight growth at 37°C. Single isolated colonies were used to inoculate sterile saline to a density of ~1.5 × 10^8^ CFU/mL. For disk diffusion, a single, sterile swab was soaked in the inoculated saline for at least 30 s, rotated to remove excess liquid, and used to inoculate a dry Mueller-Hinton agar (MHA) plate. Following plate inoculation, commercially available fosfomycin disks (Becton and Dickinson, Franklin Lakes, NJ) containing 200 μg of fosfomycin and 50 μg of G6P were placed in the center of plates. For AD, the saline suspension was diluted to 2 × 10^6^ CFU in Mueller-Hinton II broth (MHB) where 5 μL (10^4^ CFU) of the MHB suspension was placed onto a series of MHA plates supplemented with fosfomycin plus 25 μg/mL of G6P. The concentrations of fosfomycin used for AD ranged from 1 to 256 μg/mL. Enterococcus faecalis ATCC 29212 was included as a control strain and was run in parallel with test isolates for each susceptibility test.

Due to the lack of established breakpoints for K. pneumoniae and oral fosfomycin, the oral fosfomycin MIC breakpoints and all DD breakpoints for E. coli using both CLSI and EUCAST criteria and the MIC breakpoints for i.v. fosfomycin were applied as written.

### Correlation of susceptibility testing.

Correlations between CLSI AD, CLSI DD, EUCAST_oral_ AD, EUCAST_oral_ DD, and EUCAST_IV_ DD results were calculated using EUCAST_IV_ AD as the reference method. Categorical agreement was achieved when a test MIC value agreed with the interpretive criteria (susceptible/intermediate/resistant) results from the given reference method (10, 21). A minor error occurred when either a test method deemed an isolate to be intermediate while the reference method deemed the isolate to be resistant or susceptible or when a test method deemed an isolate to be susceptible or resistant and the reference method deemed it to be intermediate. A major error occurred when the reference method determined the isolate to be susceptible and the test method deemed it to be resistant. A very major error occurred when the reference method deemed the isolate to be resistant and the test method deemed it to be susceptible (10, 21).

Scattergram comparisons depicting zone diameter measurements and MIC values were constructed using R desktop version and Rstudio (Boston, MA).
